# Supplementary material for: Reduced order modeling and analysis of the human complement system
Source: PLoS One. 2017 Nov 20;12(11):e0187373. doi: 10.1371/journal.pone.0187373 (PMC5695804; doi:10.1371/journal.pone.0187373)
Supplement: S1 Table — All unlisted proteins had an initial condition of zero. (PDF) [file pone.0187373.s001.pdf]

S1 Table. Complement protein initial conditions and associated species indexed used in the model. All unlisted proteins had an initial condition of zero.

Protein	Index	Initial Condition	Units	Source
C4	2	1.9	μM	[21]
C2	3	0.322	μM	[21]
C3	8	7.57	μM	[21]
C5	1	0.195	μM	[21]
Factor H	17	2.23	μM	[21]
C4BP	18	0.417	μM	[21]
