# Supplementary material for: Reduced order modeling and analysis of the human complement system
Source: PLoS One. 2017 Nov 20;12(11):e0187373. doi: 10.1371/journal.pone.0187373 (PMC5695804; doi:10.1371/journal.pone.0187373)
Supplement: S2 Table — Two sets of C3a and C5a measurements were used for model training while three were used for validation. The information of the experimental data, its usage in this work and its location within the original publication is described below. (PDF) [file pone.0187373.s002.pdf]

S2 Table. Experimental measurements used in modeling training and validation from Morad and coworkers [35]. Two sets of C3a and C5a measurements were used for model training while three were used for validation. The information of the experimental data, its usage in this work and its location within the original publication is described below.

Protein	zymosan (μM)	Figure Number	Model Utilization
C3a	0	Fig 2a	Training
	1.0	Fig 2e	Training
	0.001	Fig 2b	Prediction
	0.01	Fig 2c	Prediction
	0.1	Fig 2d	Prediction
C5a	0	Fig 3a(i)	Training
	1.0	Fig 3c	Training
	0.001	Fig 3a(ii)	Prediction
	0.01	Fig 3a(iii)	Prediction
	0.1	Fig 3b	Prediction
