# Supplementary material for: Reduced order modeling and analysis of the human complement system
Source: PLoS One. 2017 Nov 20;12(11):e0187373. doi: 10.1371/journal.pone.0187373 (PMC5695804; doi:10.1371/journal.pone.0187373)
Supplement: S3 Table — (PDF) [file pone.0187373.s003.pdf]

S3 Table. Model species table.

Species	Index	Description
Initiator2	x[1]	Initiator complex 2, lectin pathway
C4	x[2]	C4 protein
C2	x[3]	C2 protein
C4a	x[4]	C4a protein
C4b	x[5]	C4b protein
C2a	x[6]	C2a protein
C2b	x[7]	C2b protein
C3	x[8]	C3 protein
C3b	x[9]	C3b protein
C4bC2a	x[10]	Classical/Lectin C3 convertase
C3Convertase2	x[11]	Alternative C3 convertase
C4bC2aC3b	x[12]	Classical C5 convertase
C5Convertase2	x[13]	Alternative C5 convertase
C5	x[14]	C5 protein
C5a	x[15]	C5a protein
C5b	x[16]	C5b protein
C4BP	x[17]	C4 binding protein
FactorH	x[18]	Factor H
C3a	x[19]	C3a protein
